# Retraction: Physio-biochemical responses and expressional profiling analysis of drought tolerant genes in new promising rice genotype

**DOI:** 10.1371/journal.pone.0272395

**Published:** 2022-08-17

**Authors:** The PLOS ONE Editors

The *PLOS ONE* Editors retract this article [1] because it was identified as one of a series of submissions for which we have concerns about authorship, competing interests, and peer review. We regret that the issues were not addressed prior to the article’s publication.

KAB, SF, and EAR did not agree with the retraction. HAF responded but expressed neither agreement nor disagreement with the editorial decision. All other authors either did not respond directly or could not be reached.
